# Supplementary material for: Validated LC-MS/MS Assay for the Quantitative Determination of Fenretinide in Plasma and Tumor and Its Application in a Pharmacokinetic Study in Mice of a Novel Oral Nanoformulation of Fenretinide
Source: Pharmaceutics. 2024 Mar 12;16(3):387. doi: 10.3390/pharmaceutics16030387 (PMC10974824; doi:10.3390/pharmaceutics16030387)
Supplement: Supplementary file 1 [file pharmaceutics-16-00387-s001.zip › pharmaceutics-2897720-supplementary.pdf]

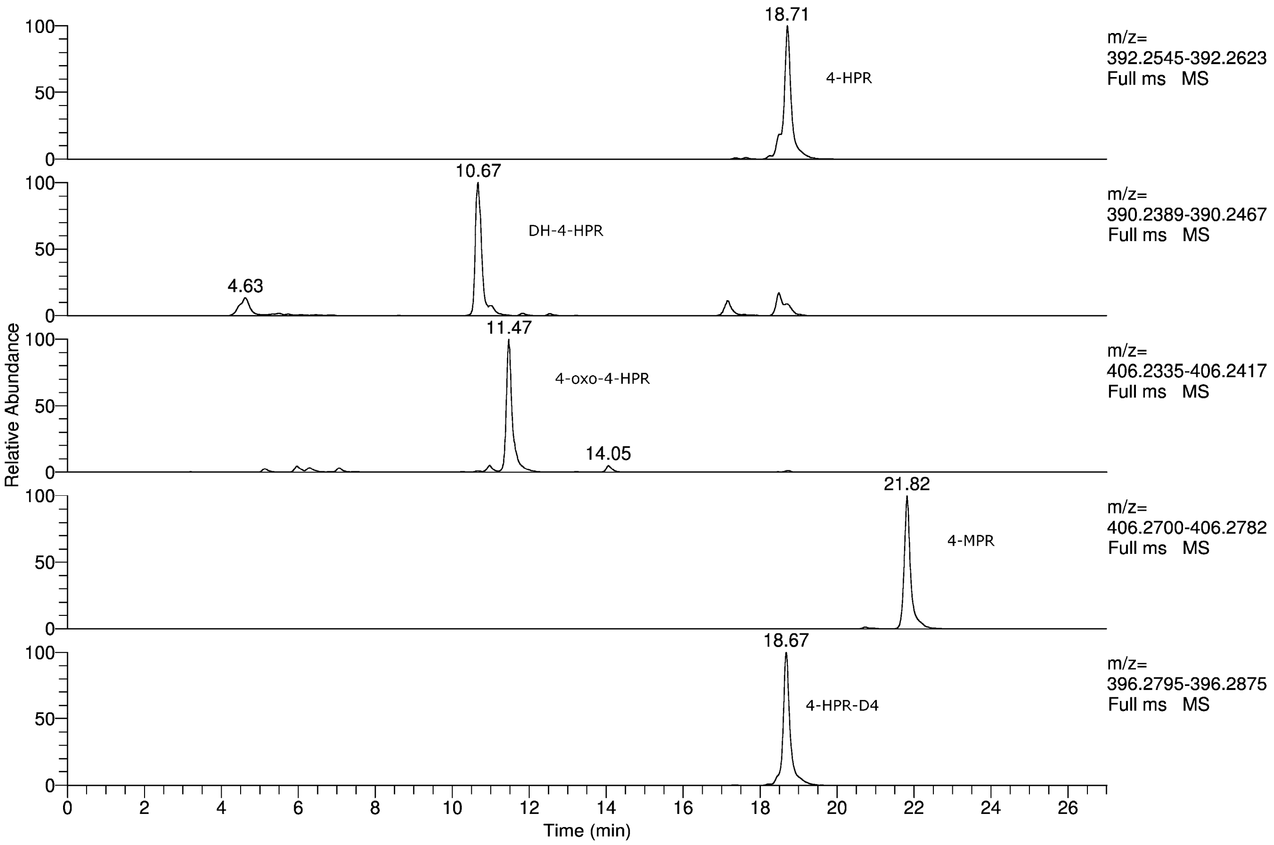

Supplementary Figure S1. High resolution ion chromatogram obtained for the m/z value (± 10 ppm) of 4-HPR and its main metabolites in a tumor sample.

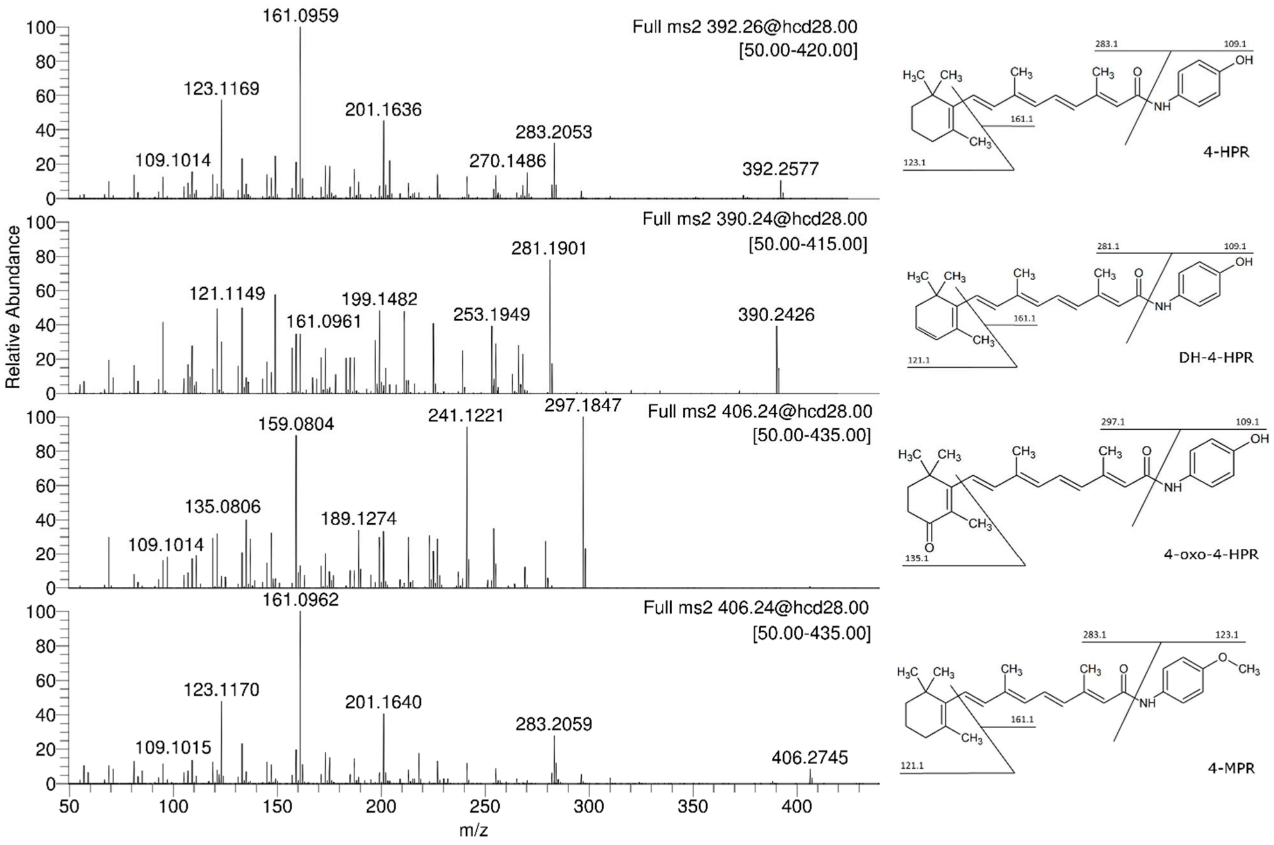

Supplementary Figure S2. MS/MS spectra of 4-HPR and its identified metabolites, highlighting some of the observed ion fragments.

Supplementary Table S1: Comparison of the characteristics of the current method with others present in the literature

	Matrices analyzed	Analytes analyzed	Common features	Differences	LOQ in plasma	Improvements
**F. Formelli et al., 1993** [12]	Plasma<br>Breast tissue	4-HPR<br>4-MPR	- Handling procedures.<br>- Chromatographic characteristics: reverse column, gradient features and run time below 10 min.	- Extractive solvent: CH_3CN<br>- HPLC-UV (λ= 340)<br>- 3 points calibration plasma range: 5-2500 ng/mL.	5 ng/ml	- Wider range of linearity applied reduces the need for dilutions.<br>- The simultaneous measurement of the analyte and the metabolites allowed a more thorough analysis of the pharmacokinetic characteristics and limited the amount of matrix needed.
**J. Vratilova et al., 2004** [13]	Tumour	4-HPR<br>4-MPR		- Extractive solvent: CH_3CN<br>- HPLC-UV (λ= 340).<br>- 6 points calibration tumor range: 0.5-20 µg/mL.	0.5 µg/mL.	
**J. I. Lee et al., 2008** [10]	Plasma	4-HPR<br>4-MPR		- Extractive solvent: CH_3CN<br>- HPLC-APCI-MS/MS. MRM +<br>- 8 points calibration plasma range: 0.5-100 ng/mL.<br>- Target ions m/z 392.4/283.3 for 4-HPR and m/z 406.3/283.2 for 4-MPR.	0.5 ng/ml	
**H.E. Cho et al., 2017** [14]	Plasma	4-HPR<br>4-MPR<br>4-oxo-4-HPR		- Extractive solvent: CH_3CH_2OH<br>- HPLC-ESI-QTRAP. MRM +<br>- 8 points calibration plasma range: 0.2-50.0 ng/mL.<br>- Target ions m/z 392.3/283.3 for 4-HPR, m/z 406.3/283.2 for 4-MPR and 406.3/297.2 for 4-oxo-4-HPR.	0.2 ng/ml	
**Present method**	Plasma<br>Tumour	4-HPR<br>4-MPR<br>4-oxo-4-HPR<br>DH-4-HPR		- Extractive solvent: CH_3CN<br>- HPLC-APCI-MS/MS. MRM +<br>- 8 points calibration plasma range: 1-500 ng/mL.<br>- 6 points calibration tumour range: 0.35-14.0 µg/g.<br>- Target ions m/z 392.4/283.3 for 4-HPR, m/z 406.3/283.2 for 4-MPR and m/z 390.2/281.1 for DH-4-HPR.	1.0 ng/ml	
